# FLASHApp: Interactive Data Analysis and Visualization for Top‐Down Proteomics

**DOI:** 10.1002/pmic.70042

**Published:** 2025-09-21

**Authors:** Tom David Müller, Jihyung Kim, Andrew Almaguer, Ayesha Feroz, Jaekwan Kim, Axel Walter, Wonhyeuk Jung, Oliver Kohlbacher, Kyowon Jeong

**Affiliations:** ^1^ Applied Bioinformatics, Department for Computer Science University of Tübingen Tübingen Germany; ^2^ Institute for Bioinformatics and Medical Informatics University of Tübingen Tübingen Germany; ^3^ jambit GmbH Stuttgart Germany; ^4^ Fullseeomics Seoul South Korea; ^5^ Clinical Pharmacology and Safety Sciences One Medimmune Way, Astrazeneca Gaithersburg Maryland USA; ^6^ Translational Bioinformatics, University Hospital Tübingen Tübingen Germany

**Keywords:** data processing and analysis, mass spectrometry, OpenMS, top‐down proteomics, web applications

## Abstract

Top‐down proteomics (TDP) is increasingly being applied in proteoform‐resolved biomedical and clinical research. However, the complexity of TDP data demands flexible visualization tools integrated with analysis workflows to streamline interpretation and validation. Existing tools lack adaptability and interactivity, often requiring researchers to invest considerable resources on additional manual processing and analysis to generate publication‐ready results and figures. This added layer of manual intervention impacts reproducibility, posing a significant challenge to consistent scientific outcomes. FLASHApp addresses these challenges by offering a web‐based, platform‐independent application for TDP data analysis and visualization. It integrates key tools like FLASHDeconv, featuring automated processing, interactive publication‐ready visualizations, and direct team collaboration via shareable URLs. FLASHApp is open‐source software as part of OpenMS and available at https://www.openms.org/FLASHApp/.

1

Top‐down proteomics (TDP) based on mass spectrometry (MS) is being adopted in a broader range of proteoform resolved biomedical studies and clinical research [1, 2]. While the method has been generating many mechanistic biological insights [3, 4, 5], including cancer biology [6], new TDP protocols are still being introduced and tested for further enhancement in all areas of proteoform analysis including identification [7, 8] and quantification [9] with variety of technical and experimental breakthroughs both in denatured [10] and native proteomics approaches [11].

The complexity of TDP data analysis workflows demands flexible, integrated, and interactive software solutions that handle both analytical processes and visualization of results. However, existing tools often lack either the flexibility to deal with the many different experimental protocols or the user‐friendliness. Users thus spend considerable time performing manual tasks, such as transferring data between multiple software platforms or conducting detailed manual inspections of MS spectra. These manual interventions are not only time‐ and resource‐intensive but also introduce variability, decrease reproducibility, and ultimately pose challenges to achieving consistent and reliable scientific outcomes. An integrated workflow platform that seamlessly combines data processing and visualization can help address these challenges.

Existing TDP analysis software suites such as ProSightPD [12], Prosight Native [13] (which incorporates TDValidator), Proteoform Suite [14], Informed‐Proteomics [15], and MASH Explorer [16] offer integrated visualization modules, significantly enhancing data interpretation and result presentation. In addition to these platforms, several visualization focused software tools have been developed. For instance, TopMSV [17] is a web‐based tool designed for visualizing TDP‐MS data that integrates TopPIC [7] tools. As an open‐source software, TopMSV streamlines the workflow from raw data to final analytical results. Other notable open‐source platforms include TopDownApp [18] and PSpecteR [19], which have visualization and analysis capabilities specifically tailored for TDP data.

From a user perspective, however, existing dashboards designed for viewing results, although informative, typically have rigid content structures and layouts predetermined by developers. This rigidity significantly limits their adaptability to accommodate diverse TDP protocols. Comprehensive data analysis, therefore, often necessitates reliance on additional handcrafted scripts and third‐party tools such as ThermoFisher Freestyle for spectral visualization and ProSight Lite [20] for fragment ion annotation. The absence of integration prevents tools from efficiently identifying relevant data, requiring users to transfer the data between different tools and to manually curate and filter information. Furthermore, most proprietary software remains neither open‐source nor freely available, restricting access and hindering the reliable reproducibility of results. Moreover, web‐based solutions continue to face deployment challenges.

Here we present FLASHApp, an open‐source, platform‐independent web application for interactive analysis and visualization of TDP‐MS datasets. FLASHApp provides an end‐to‐end solution for data analysis and visualization, with full user control over the layout and presentation of visual components. It is built on the OpenMS web application template [21] and currently integrates complete workflows for spectral deconvolution using FLASHDeconv [22] and for proteoform identification and characterization using FLASHTnT (manuscript under preparation). It includes specialized and interactive visualizations tailored to TDP‐MS, such as annotation of proteoforms by terminal and internal fragment ions, and a 3D representation of signal and noise peaks for deconvolved masses. All visualization components can be freely arranged in a customizable grid, with the option to view multiple experiments side‐by‐side, and exported as publication‐ready figures in SVG format.

FLASHApp is accessible via any modern web browser, independent of the operating system and hardware requirements. It is publicly available on the OpenMS Website (www.openms.org/FLASHApp/) and can easily be hosted in‐house (e.g., by core facilities) via a containerized image (the corresponding Dockerfile is freely available under a permissive three‐clause BSD license). Additionally, a Windows installer enables offline use, particularly benefiting wet lab scientists without a strong computational background.

The web application is organized into multiple sections, which can be navigated through a sidebar (see Figure 1). Each section consists of multiple pages. The first section serves as the entry point, offering a quickstart page, alongside a comprehensive documentation page that provides detailed, user‐oriented explanations and guidance on using FLASHApp. Each of the subsequent sections focuses on a specific workflow, consisting of standardized, user‐friendly pages and page elements.

**FIGURE 1:**
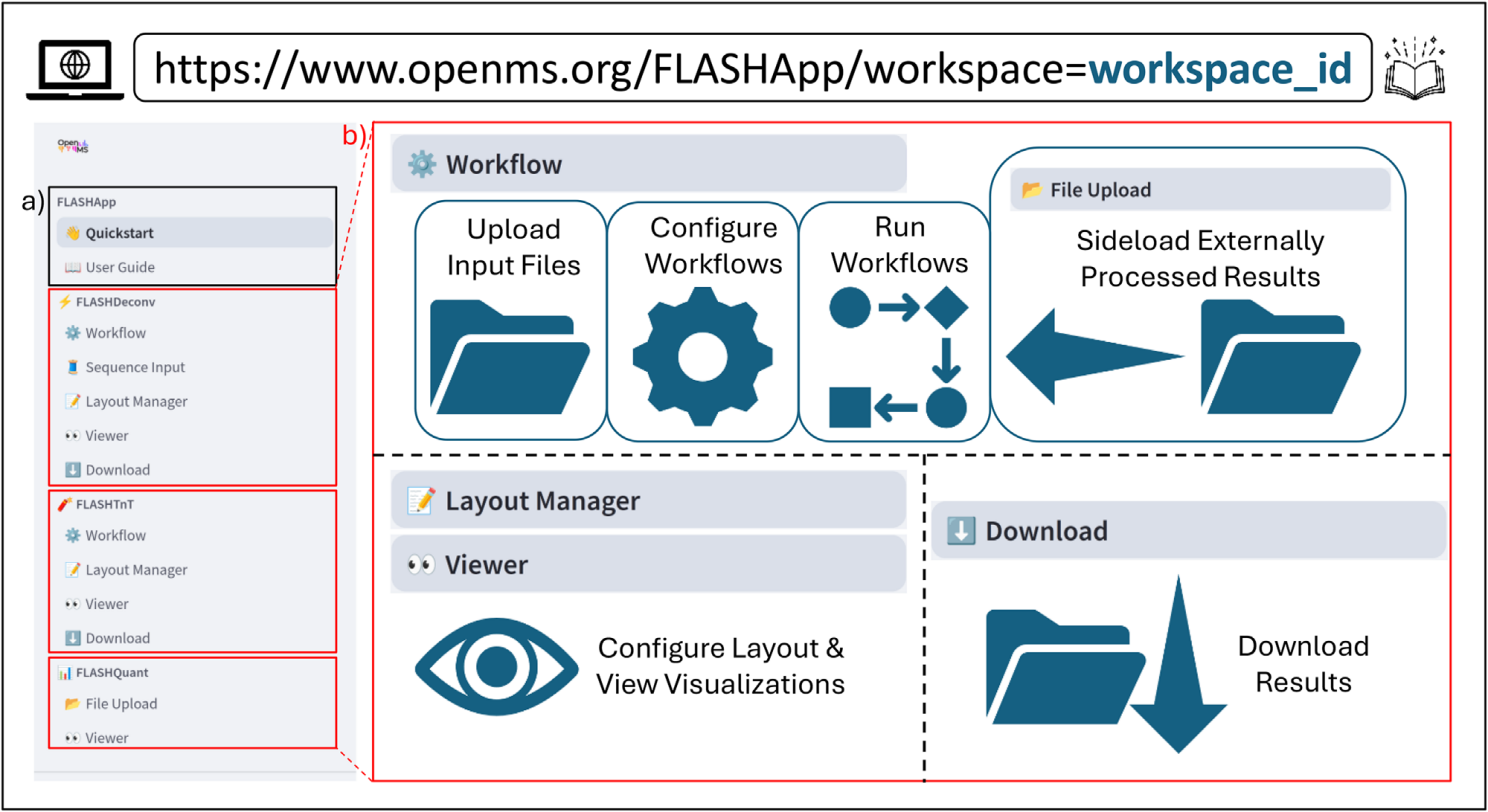
FLASHApp is structured into multiple sections. The first section (a) provides comprehensive, user‐oriented documentation to assist new users. Subsequent sections (b) correspond to distinct workflows, enabling users to upload input files, configure the processing pipeline, execute workflows, and download results. Dedicated pages for layout management and result visualization allow users to customize dashboards and conduct detailed analysis tailored to specific research needs.

At the time of publication, workflows for FLASHDeconv and FLASHTnT are fully integrated within FLASHApp, enabling users to seamlessly perform spectral deconvolution, proteoform identification, and characterization entirely within the application. These workflows are accessible through Workflow pages located within the respective sections. Workflow pages are designed to guide users systematically through uploading input files, configuring analysis parameters, and executing the workflows.

A workflow page contains three basic tabs. First, the Input File Upload tab facilitates uploading raw data files required for processing. For more efficient batch processing, FLASHApp also allows uploading entire folders of data rather than individual files when running locally. Second, the Configuration tab allows users to select input files for analysis and conveniently configure individual tool parameters within the workflow. Parameter settings can be imported or exported in a user‐friendly, human‐readable format. To enhance user experience, the interface by default presents only the most relevant parameters, while less frequently used (advanced) parameters can be accessed via a toggle. Lastly, from the Run tab, workflows can be executed with a single click of the Start button. Input file processing is handled automatically, and users receive real‐time status updates through a live log view. The log display is customizable to suit different user groups and preferences. By default, a minimal log, designed for non‐technical users, provides a clear, natural‐language summary of the current processing stage. Alternatively, users can enable more detailed views that display command execution details, runtimes, or even the full output of each tool. In addition, FLASHApp automatically generates a comprehensive method summary, including citations of individual tools along with details of any modified parameters.

To ensure reproducibility and facilitate project management, workflow input and output data are stored in dedicated workspaces. In online mode, a new workspace is automatically created upon entering FLASHApp and is embedded in the website URL. This allows users to bookmark and revisit their analysis anytime. Shareable URLs further enhance collaboration by enabling team members to access and contribute to the same workspace. In offline mode, users can create and manage workspaces at their convenience, ensuring structured organization across multiple projects. Results can be exported for further analysis using a dedicated Download page within the relevant section. These results include not only all output files from the tools used but also a human‐readable summary of the parameters applied to maximize reproducibility.

FLASHApp also supports manual upload of externally processed input files, integrated as a Manual Result Upload tab into each Workflow page. The interface displays a user‐friendly table summarizing uploaded experiments and indicates the presence or absence of input files for each experiment. Users can also manage uploaded files directly, including the option to delete experiments as necessary. This feature also enhances extensibility by allowing integration of tools that are still under development or not yet suitable for full workflow integration. A good example is FLASHQuant [9], a label‐free quantification tool, which is not currently integrated as a complete workflow but whose results can still be uploaded and analyzed within FLASHApp.

A dedicated Viewer page is assigned to each interfaced workflow, featuring visualization components specifically tailored to its functionality. For spectral deconvolution, for example, components such as MS1/2 raw/deconvolved spectrum plots and feature heatmaps dissect spectral features, while visualizations for proteoform identification and characterization include proteoform sequences, annotated with mass shifts and sequence tags. Quantification visualizations provide insights into feature detection and the resolution of conflicts. Many visualizations are interactive, enabling alterations such as adjusting fragment ion matching tolerances.

The layout of the Viewer pages can be customized on the Layout Manager pages (see Figure 2b). It allows users to configure the number of samples to display, the visualization components, and their arrangement in a grid. This flexibility could also facilitate comparative analysis, such as assessing technical replicates. Users can assign specific samples to different locations on the Viewer page and choose an individual set of visualization components for each sample. Additionally, layout configurations can be imported and exported, making it easy to share setups with collaborators and reuse them across experiments.

**FIGURE 2:**
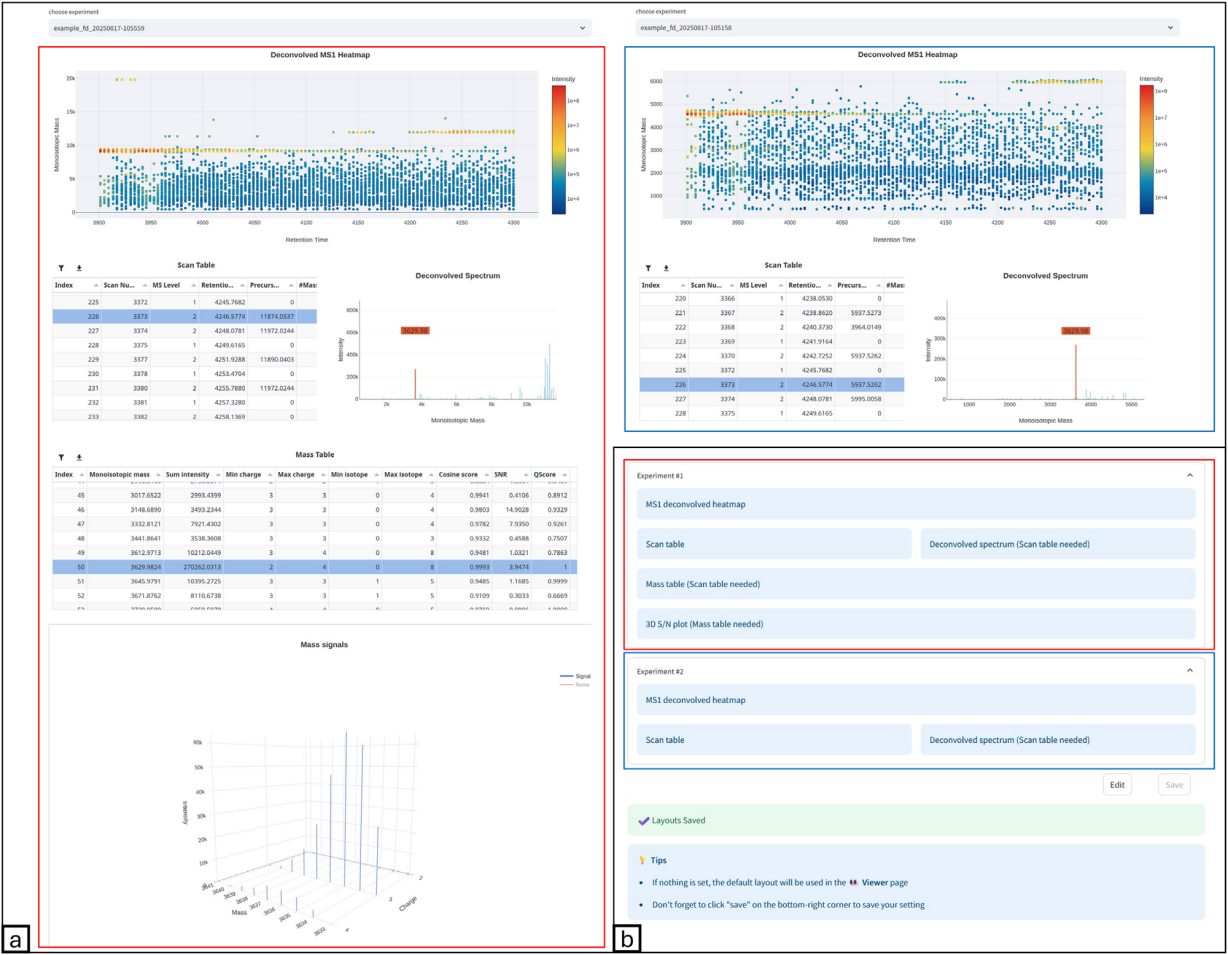
Example configuration of FLASHApp visualizations to compare deconvolution parameters. (a) Side‐by‐side comparison of deconvolution results from a single dataset using two different charge state constraints: a broad range up to +100 (left) and a narrower range up to +5 (right). (b) Layout configuration of the viewer interface.

The configurable layout allows users to tailor the visualization to their unique needs (e.g., targeted vs. discovery analysis). It is especially advantageous in TDP, where manual validation still remains prevalent. Achieving this flexibility relies on modularized visualization components that users can selectively choose and arrange according to their preferences. This modular design streamlines the implementation of future components—making FLASHApp readily extensible. To ensure a low barrier of entry for new users, a sensible default layout with a carefully selected set of components is used by default. A complete list of available component options at the time of writing is shown in Table 1. A part of visualizations support cross‐component interactivity, allowing intuitive exploration of analysis results. For instance, the scan table has the same functionality throughout many components within the viewer. By clicking on a row in the scan table, the selected scan can update components, including the mass table (showing per‐scan information), and raw/deconvolved spectra plots. Selecting a mass in any component highlights that mass and its corresponding charge states in the raw and deconvolved spectra plots. Furthermore, advanced visualizations such as the Sequence View (Figure S1) and Internal Fragment Map (Figure S3) offer interactive customization options, accessible via the gear icon in the top‐right corner. This feature empowers researchers to tailor visualizations to their specific needs.

**TABLE 1:** Available visualization components. The dependency column indicates which components are required for the corresponding component for cross component interactivity.

**Component name**	**Dependency**	**Description**
MS1/2 Raw Heatmap	—	Displays raw MS signals as a 2D heatmap with m/z on the y‐axis, retention time on the x‐axis, and intensity as a color gradient.
MS1/2 Deconvolved Heatmap	—	Displays deconvolved MS signals as a 2D heatmap with monoisotopic mass on the y‐axis, retention time on the x‐axis, and summed intensity across all charge states and isotopologues as a color gradient.
Score Distribution Plot	—	Displays the distribution of scores for deconvolved target and decoy (if reporting is enabled) masses or proteins, enabling the selection of thresholds to select high‐confidence hits.
Scan Table	—	Lists scan details (e.g., number, retention time, precursor mass).
Proteoform Table	—	Lists proteins identified by FLASHTnT, including accession, modifications, and score.
Mass Feature Table	—	Displays monoisotopic mass, retention time, and quantification for identified mass features.
Mass Table	Scan table	Details deconvolved masses for a selected scan. Displayed properties include intensity, S/N ratio, and charge/isotope range.
Tag Table	Proteoform table	Lists sequence tags used in FLASHTnT for identifying proteins, along with scores.
Raw Spectrum	Scan table	Plots raw spectrum for a selected scan (intensity vs. m/z).
Deconvolved Spectrum	Scan table	Plots the deconvolved spectrum for a scan (summed intensity vs. monoisotopic mass).
3D Signal to Noise Plot	Mass table	Visualizes S/N ratio of deconvolved masses in 3D (charge, m/z, intensity). Highlights signal and noise separately.
Sequence View	Scan table or Proteoform table	Annotates sequence tags, PTMs, and fragments. Visualizes FLASHTnT results from the Proteoform Table; For FLASHDeconv, data must be supplied by the user.
Internal Fragment Map	Scan table	Shows internal fragment ions from the selected scan. Compatible with FLASHDeconv and FLASHTnT data.
Sequence Tag View	Tag table	Overlays selected tags in raw and deconvolved spectra. Clicking a mass shows evidence in the raw spectrum.
Feature 3D Plot	Feature table	3D plot of features (charge, retention time, intensity). Feature selection is driven by the feature table. Conflicts in quantification can also be visualized.

Figure 2a presents an example configuration of the FLASHDeconv Viewer page, illustrating a side‐by‐side comparison of deconvolution results from a single dataset (deposited in MassIVE with accession MSV000091923; see data availability statement) using two different charge state constraints: a broad range up to +100 (left) and a narrower range up to +5 (right). The layout configuration is shown in Figure 2b. The heatmaps prominently highlight differences in the distribution of deconvolved masses across retention time, offering an intuitive visualization of how charge state constraints influence mass detection. The scan table, coupled with the deconvolved spectrum plot, allows users to explore deconvolved signals in detail on a per‐scan basis, clearly showing the reduction of identified masses in the selected scan when the number of considered charge states is reduced. In the left configuration, the mass table summarizes key attributes of each monoisotopic mass, including charge and isotope ranges, summed intensity, and scoring metrics. The 3D signal plot further enhances interpretability by visualizing the charge state contributions to individual masses, offering a means for direct manual validation of deconvolution outcomes.

Visualization of proteoform sequence coverage and fragment ions is popularly demanded for MS/MS analysis. Within FLASHApp, this demand is addressed by a variety of components. First, the Sequence View component (see Figure S1) visualizes potential matches between theoretical fragment masses, derived from a given protein sequence in ProForma notation [23], and deconvolved fragment masses. For FLASHDeconv workflows, it supports targeted proteoform characterization, allowing users to specify a proteoform either directly in ProForma notation or through an interactive interface, where fixed and variable modifications can be defined and explored in real‐time.

For automatic proteoform identification and characterization in FLASHTnT, the Sequence View component also supports the visualization of truncations, mass shifts with ambiguous localization, and sequence tags, enhancing the interpretability of results. Once an MS2 scan or identified proteoform is selected, the Sequence View dynamically updates to display the corresponding matches, ensuring seamless and interactive data exploration.

Users can tailor the sequence view as needed by clicking the gear icon in the upper right corner of the sequence map, adjusting key parameters, such as fragment ion types and mass tolerance. Clicking the “i” icon next to the gear icon will inform users what each symbol on the sequence map means. For targeted annotation in FLASHDeconv workflows, a right‐click on a residue cell within the sequence map opens a pop‐up menu, enabling the addition of post‐translational modification (PTM) mass shifts to a user‐provided sequence. A few pre‐defined PTM sets for each amino acid type are available, and users can also include custom mass shifts. By clicking on the residue cell with a marker indicating a matched fragment, the associated rows in the mass table and matching fragment table are highlighted. This helps users to review the detailed information easily.

Figure S2 shows an example viewer page for a proteoform identification workflow with FLASHTnT. The proteoform table allows the selection of different hits from the preceding search. Selection of entries in the proteoform table automatically updates the sequence view. The tag table displays all sequence tags that are associated with the selected hit.

All visualization components in FLASHApp support interactive features such as dragging, zooming, and, for 3D plots, rotation. Additionally, all visualizations (including the sequence view) can be exported in an SVG format. Tables can be sorted and filtered by columns and exported as CSV files. This functionality not only allows scientists to generate publication‐ready figures directly within FLASHApp but also facilitates effortless further analysis of the data.

FLASHApp is based on the OpenMS web application template [21] and is developed in Python and TypeScript. The web framework Streamlit (version 1.43.0) is used for front‐end and back‐end development. The OpenMS web application template ensures OpenMS [24] TOPP tools such as FLASHDeconv [22], FLASHTnT, and third‐party tools can readily be configured and executed, easing future extension with additional workflows and tools. The results and input data are processed using common Python libraries such as pyOpenMS [25] and Pandas [26]. Visualizations are created using Vue.js with the packages Plotly.js, Tabulator, and Vuetify. They are embedded in Streamlit as custom components.

FLASHApp runs on a Streamlit server and can be deployed either locally or online. For local execution, users can choose between two convenient options: running the publicly available source code or using a pre‐packaged Windows installer. For online hosting scenarios (e.g., by core facilities), a containerized Docker image is provided. Additionally, OpenMS offers an online version of FLASHApp, freely accessible at https://www.openms.org/FLASHApp/. The source code is available at https://github.com/OpenMS/FLASHApp.

In summary, FLASHApp provides a powerful and flexible open‐source platform for automated processing, intuitive visualization, and interactive exploration of TDP data, addressing the critical need for accessible and versatile analysis tools in proteomics research. Its modular and open‐source architecture enables streamlined and community‐driven extension with new workflows and additional customized visualization components tailored precisely to diverse experimental setups and analytical objectives, enabling the platform to keep pace with the rapid evolution of the TDP field.

A key feature of FLASHApp is its highly configurable layout, allowing users to selectively arrange visualization modules specifically suited to their experimental needs. Users can dynamically customize views for simultaneous comparison of multiple datasets. For example, researchers can easily display complex heatmaps, detailed spectrum annotations, and informative tables side by side, simplifying manual validation and thorough exploration, which are essential steps in current TDP workflows.

The layout customization not only enhances usability but also significantly broadens FLASHApp's application range. Users can create optimized visual configurations tailored precisely to their research goals, effectively minimizing computational and cognitive overhead by incorporating only necessary visualization components. Importantly, these tailored visual setups can be readily exported and shared using the website URL, facilitating collaboration and reproducibility across labs. By combining user‐friendly design, powerful interactive visualization capabilities, and ease of integration and extension, FLASHApp effectively addresses current limitations in TDP data analysis, promoting collaboration and innovation within the proteomics research community.

In future developments, we plan to extend FLASHApp with additional workflows. OpenMS TDP tools such as FLASHQuant and FLASHIda [8] will be fully integrated into FLASHApp, streamlining quantitative analyses and enabling workflows synergistic with intelligent data acquisition strategies. We also aim to incorporate further visualizations, including signal and isotope peak annotations across retention time for user‐defined masses. It is noteworthy that while FLASHApp is built upon the OpenMS web application template, it is not limited to OpenMS tools—the modular architecture supports integration of third‐party tools (i.e., arbitrary executables can be integrated in workflows). As such, FLASHApp is poised to become a uniquely powerful, accessible, and extensible platform that meets the evolving needs of the TDP community.

## Author Contributions

T.D.M. and Ji.K. contributed equally. Ji.K., W.J., K.J., and O.K. conceived the work. T.D.M., Ji.K., A.A., A.F., Ja.K., and A.W. implemented the software. T.D.M., Ji.K., and K.J. wrote the manuscript. All authors read and approved the final manuscript.

## Conflicts of Interest

The authors declare the following competing financial interest(s): Oliver Kohlbacher is an officer in OpenMS Inc., a non‐profit foundation that manages the international coordination of OpenMS development. All remaining authors declare no competing interests.

## Supporting information




**Supporting File**: pmic70042‐sup‐0001‐SuppMat.docx

## Data Availability

The dataset used as an example for the figures is openly available in the MassIVE repository (ID: MSV000091923) at DOI 10.25345/C5XW4861V. FLASHApp is open‐source software as part of OpenMS. All FLASHApp source code is available on GitHub (https://github.com/OpenMS/FLASHApp) under a permissive three‐clause BSD license.
