# Supplementary material for: FLASHApp: Interactive Data Analysis and Visualization for Top‐Down Proteomics
Source: Proteomics. 2025 Sep 21;25(24):15–21. doi: 10.1002/pmic.70042 (PMC12716109; doi:10.1002/pmic.70042)
Supplement: Supplementary file 1 — Supporting File: pmic70042‐sup‐0001‐SuppMat.docx [file PMIC-25--s001.docx]

Final Draft

# Supplementary


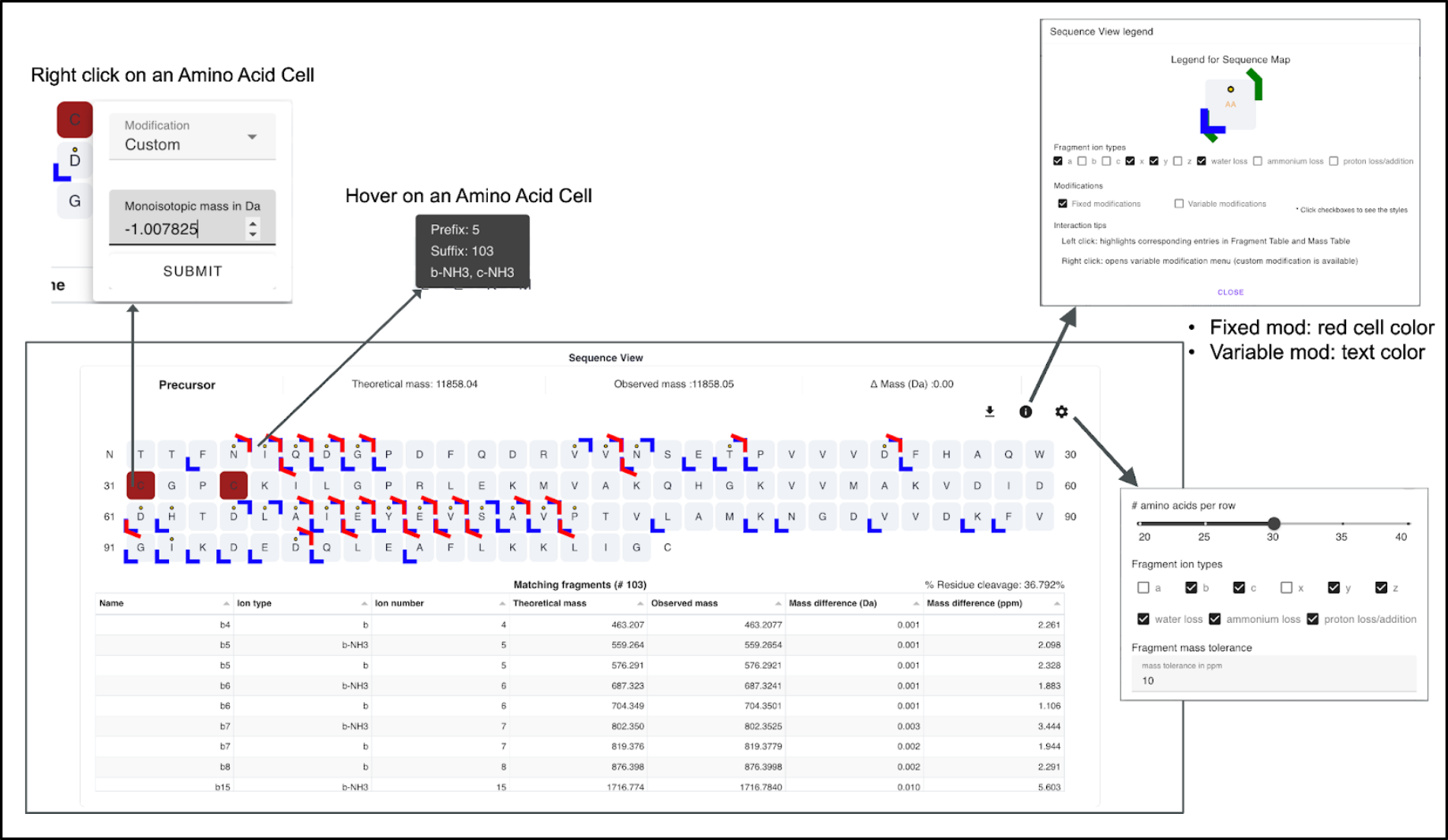


**Supplementary Figure 1:** An example screenshot of the Sequence View component. It demonstrates how the component is displayed on the Viewer page and the different functionalities of the sequence view. A truncated sequence of the Human Thioredoxin protein is matched to a deconvolved MS2 scan from the same data used in Supplementary Fig. 3. The red-colored cells on the sequence indicate that variable modifications have been added to them. Different fragment types are colored in distinctive colors: green for a/x, blue for b/y, and red for c/z. In this example, the b/y and c/z ion types have been selected (panel on the bottom-right), so the blue and red markers are shown on the sequence. Yellow dots on the residue cell imply that the fragment masses have been matched to the theoretical masses after water loss, ammonium loss, or proton loss/addition. To check this information, users can click on the "i" icon on the top-right corner of the sequence view to open the legend menu (top-right panel on the figure).


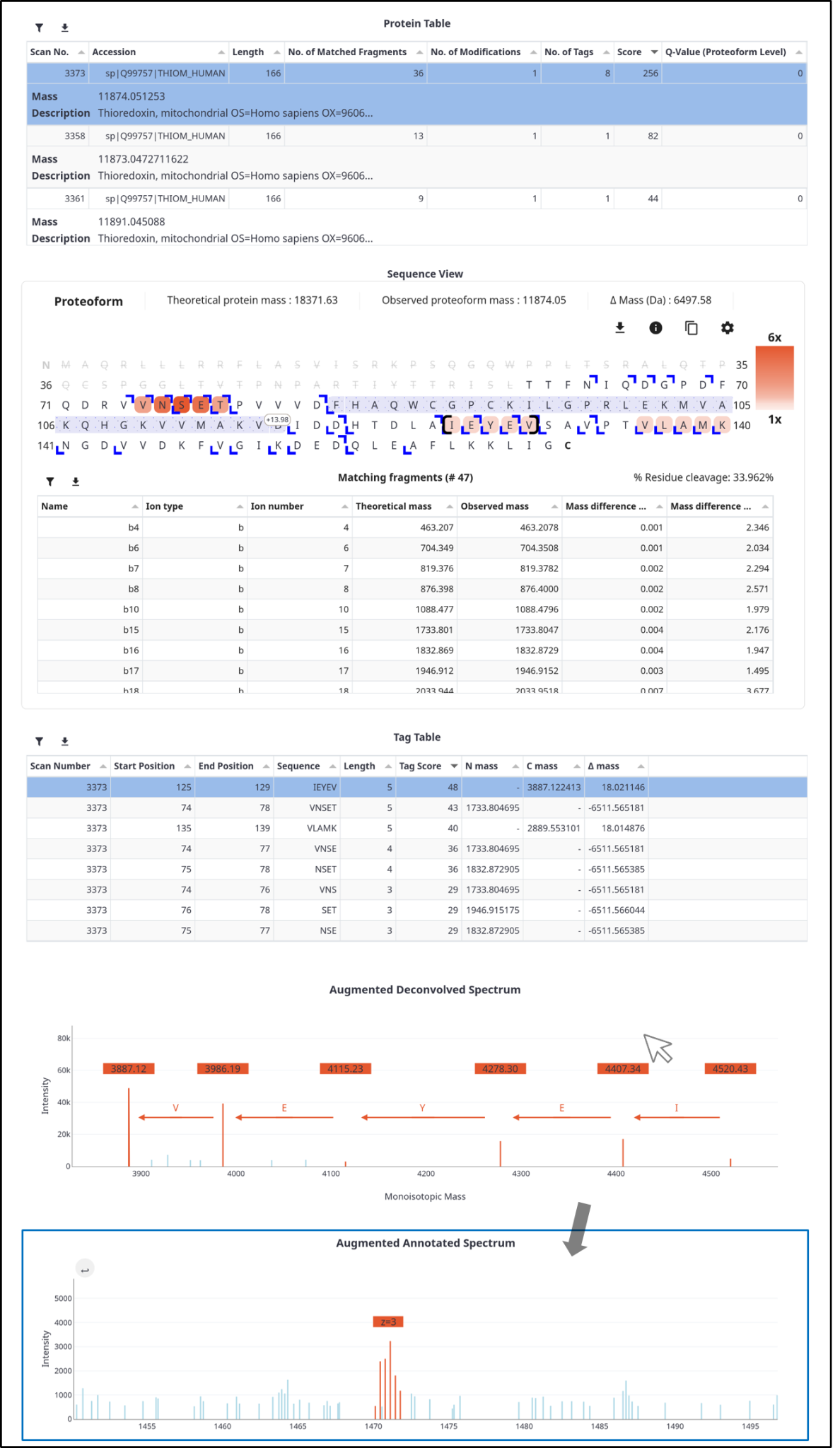


**Supplementary Figure 2:** Example viewer page from a proteoform identification workflow using FLASHTnT. The Proteoform Table lists the identified proteoforms; selecting a proteoform updates both the Sequence View and the Tag Table. In FLASHTnT workflows, the Sequence View is automatically extended to show sequence tag coverage (orange gradient) and ambiguously assigned PTMs (blue). The Tag Table displays the corresponding sequence tags, which are highlighted in the Sequence View with black borders when selected. Each tag can be inspected within an annotated deconvolved spectrum. Clicking on a tag mass in the deconvolved spectrum reveals the supporting evidence in the raw spectrum (blue box).


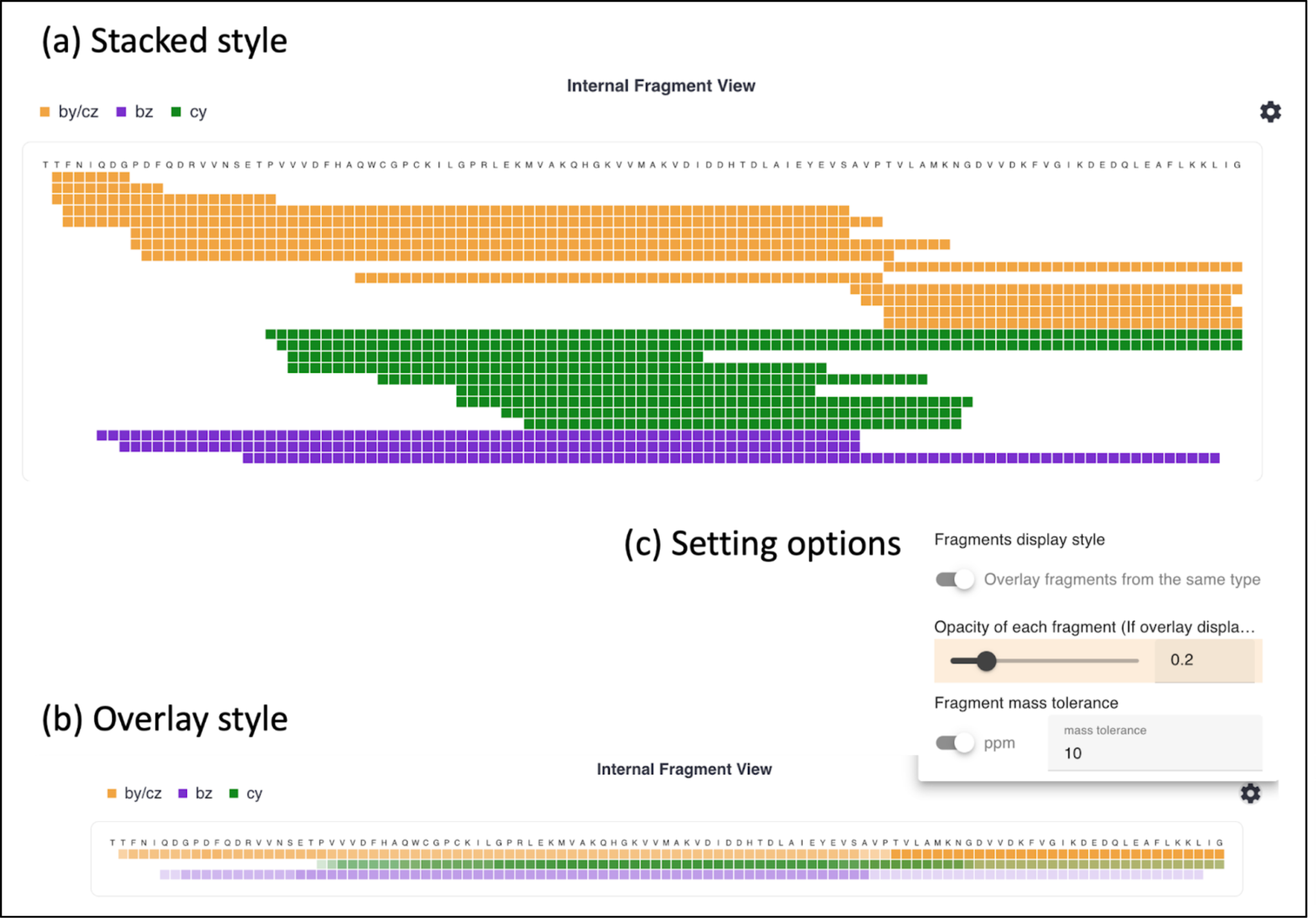


**Supplementary Figure 3:** An example screenshot of the internal fragment map component. This component has two types of visualizing internal fragments: *(a)* Stacked and *(b)* Overlay. Two types can be switched with the toggle button in the settings menu in *(c)*.
